# Supplementary material for: Functional Profiling of Soft Tissue Sarcoma Using Mechanistic Models
Source: Int J Mol Sci. 2023 Sep 29;24(19):14732. doi: 10.3390/ijms241914732 (PMC10572617; doi:10.3390/ijms241914732)
Supplement: Supplementary file 1 [file ijms-24-14732-s001.zip › figure S2.pdf]

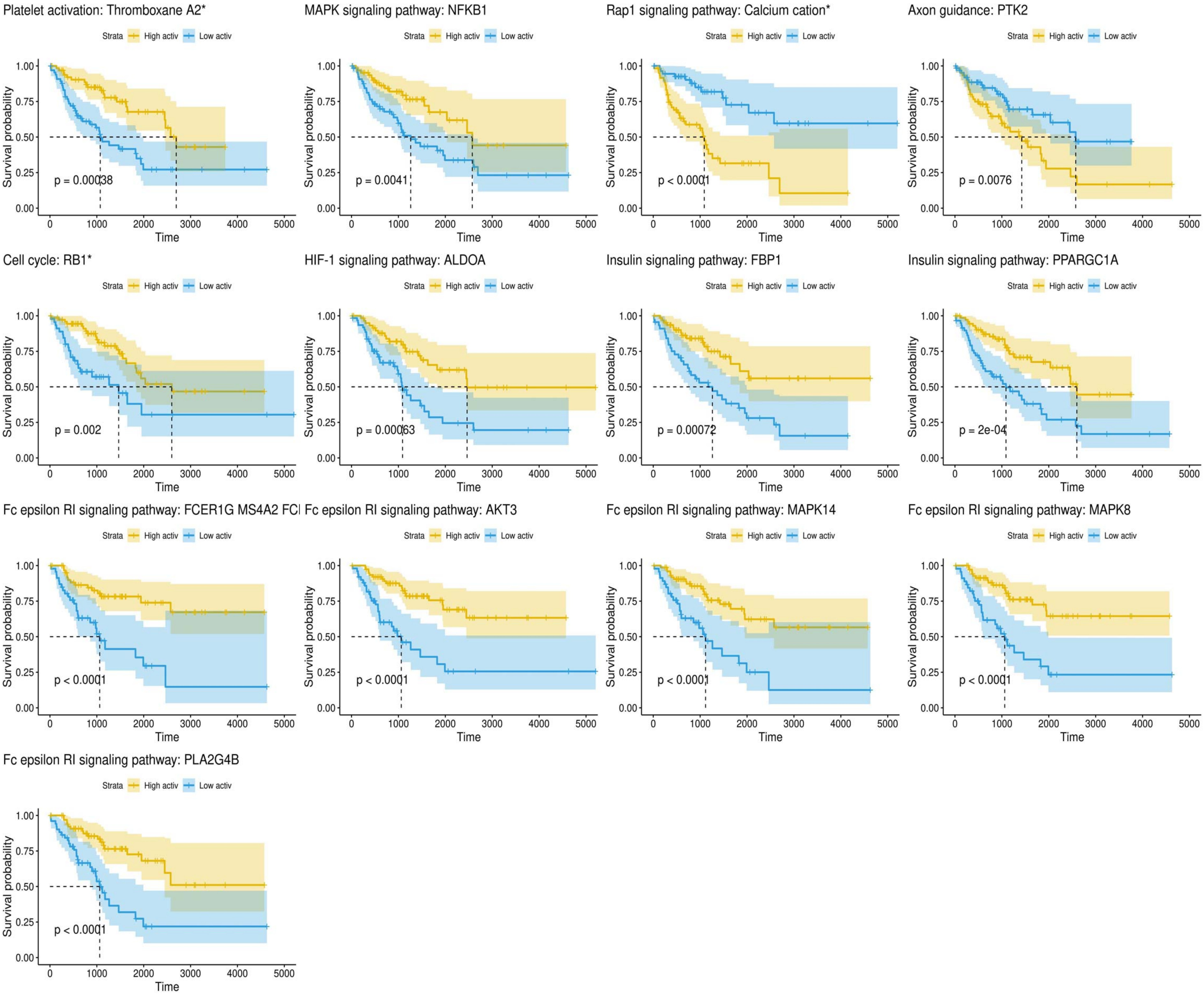
Platelet activation: Thromboxane A2*
Strata High activ Low activ
Survival probability
Time
p = 0.00038
MAPK signaling pathway: NFKB1
Strata High activ Low activ
Survival probability
Time
p = 0.0041
Rap1 signaling pathway: Calcium cation*
Strata High activ Low activ
Survival probability
Time
p < 0.0001
Axon guidance: PTK2
Strata High activ Low activ
Survival probability
Time
p = 0.0076
Cell cycle: RB1*
Strata High activ Low activ
Survival probability
Time
p = 0.002
HIF-1 signaling pathway: ALDOA
Strata High activ Low activ
Survival probability
Time
p = 0.00063
Insulin signaling pathway: FBP1
Strata High activ Low activ
Survival probability
Time
p = 0.00072
Insulin signaling pathway: PPARGC1A
Strata High activ Low activ
Survival probability
Time
p = 2e-04
Fc epsilon RI signaling pathway: FCER1G MS4A2 FCI
Strata High activ Low activ
Survival probability
Time
p < 0.0001
Fc epsilon RI signaling pathway: AKT3
Strata High activ Low activ
Survival probability
Time
p < 0.0001
Fc epsilon RI signaling pathway: MAPK14
Strata High activ Low activ
Survival probability
Time
p < 0.0001
Fc epsilon RI signaling pathway: MAPK8
Strata High activ Low activ
Survival probability
Time
p < 0.0001
Fc epsilon RI signaling pathway: PLA2G4B
Strata High activ Low activ
Survival probability
Time
p < 0.0001
