# Supplementary material for: Functional Profiling of Soft Tissue Sarcoma Using Mechanistic Models
Source: Int J Mol Sci. 2023 Sep 29;24(19):14732. doi: 10.3390/ijms241914732 (PMC10572617; doi:10.3390/ijms241914732)
Supplement: Supplementary file 1 [file ijms-24-14732-s001.zip › figure S3.pdf]

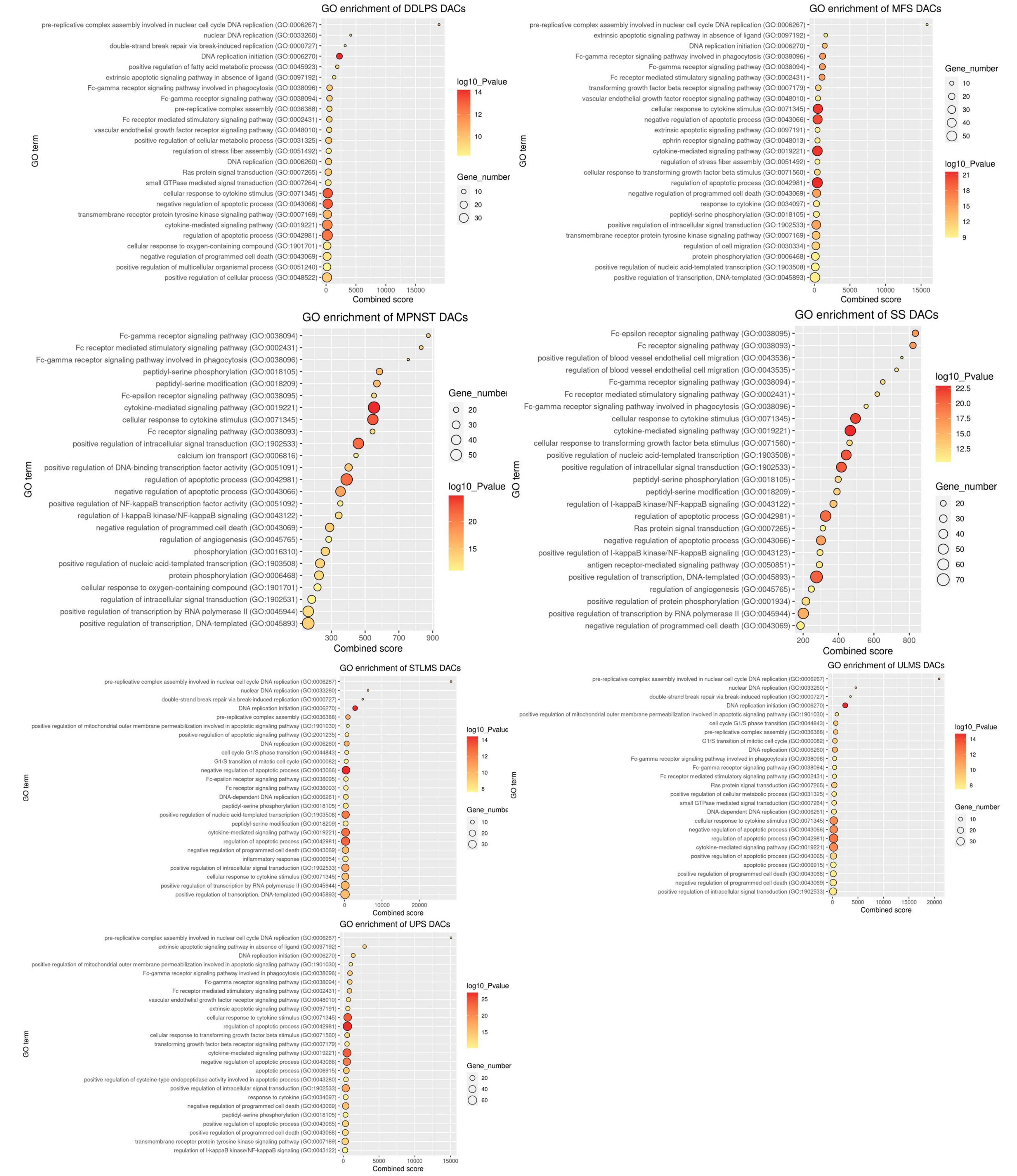
GO enrichment of DDLPS DACs
pre-replicative complex assembly involved in nuclear cell cycle DNA replication (GO:0006267)
nuclear DNA replication (GO:0033260)
double-strand break repair via break-induced replication (GO:0000727)
DNA replication initiation (GO:0006270)
positive regulation of fatty acid metabolic process (GO:0045923)
extrinsic apoptotic signaling pathway in absence of ligand (GO:0097192)
Fc-gamma receptor signaling pathway involved in phagocytosis (GO:0038096)
Fc-gamma receptor signaling pathway (GO:0038094)
pre-replicative complex assembly (GO:0036388)
Fc receptor mediated stimulatory signaling pathway (GO:0002431)
vascular endothelial growth factor receptor signaling pathway (GO:0048010)
positive regulation of cellular metabolic process (GO:0031325)
regulation of stress fiber assembly (GO:0051492)
DNA replication (GO:0006260)
Ras protein signal transduction (GO:0007265)
small GTPase mediated signal transduction (GO:0007264)
cellular response to cytokine stimulus (GO:0071345)
negative regulation of apoptotic process (GO:0043066)
transmembrane receptor protein tyrosine kinase signaling pathway (GO:0007169)
cytokine-mediated signaling pathway (GO:0019221)
regulation of apoptotic process (GO:0042981)
cellular response to oxygen-containing compound (GO:1901701)
negative regulation of programmed cell death (GO:0043069)
positive regulation of multicellular organismal process (GO:0051240)
positive regulation of cellular process (GO:0048522)
GO term
0 5000 10000 15000
Combined score
log10_Pvalue
14 12 10
Gene_number
10 20 30
GO enrichment of MFS DACs
pre-replicative complex assembly involved in nuclear cell cycle DNA replication (GO:0006267)
extrinsic apoptotic signaling pathway in absence of ligand (GO:0097192)
DNA replication initiation (GO:0006270)
Fc-gamma receptor signaling pathway involved in phagocytosis (GO:0038096)
Fc-gamma receptor signaling pathway (GO:0038094)
Fc receptor mediated stimulatory signaling pathway (GO:0002431)
transforming growth factor beta receptor signaling pathway (GO:0007179)
vascular endothelial growth factor receptor signaling pathway (GO:0048010)
cellular response to cytokine stimulus (GO:0071345)
negative regulation of apoptotic process (GO:0043066)
extrinsic apoptotic signaling pathway (GO:0097191)
ephrin receptor signaling pathway (GO:0048013)
cytokine-mediated signaling pathway (GO:0019221)
regulation of stress fiber assembly (GO:0051492)
cellular response to transforming growth factor beta stimulus (GO:0071560)
regulation of apoptotic process (GO:0042981)
negative regulation of programmed cell death (GO:0043069)
response to cytokine (GO:0034097)
peptidyl-serine phosphorylation (GO:0018105)
positive regulation of intracellular signal transduction (GO:1902533)
transmembrane receptor protein tyrosine kinase signaling pathway (GO:0007169)
regulation of cell migration (GO:0030334)
protein phosphorylation (GO:0006468)
positive regulation of nucleic acid-templated transcription (GO:1903508)
positive regulation of transcription, DNA-templated (GO:0045893)
GO term
0 5000 10000 15000
Combined score
Gene_number
10 20 30 40 50
log10_Pvalue
21 18 15 12 9
GO enrichment of MPNST DACs
Fc-gamma receptor signaling pathway (GO:0038094)
Fc receptor mediated stimulatory signaling pathway (GO:0002431)
Fc-gamma receptor signaling pathway involved in phagocytosis (GO:0038096)
peptidyl-serine phosphorylation (GO:0018105)
peptidyl-serine modification (GO:0018209)
Fc-epsilon receptor signaling pathway (GO:0038095)
cytokine-mediated signaling pathway (GO:0019221)
cellular response to cytokine stimulus (GO:0071345)
Fc receptor signaling pathway (GO:0038093)
positive regulation of intracellular signal transduction (GO:1902533)
calcium ion transport (GO:0006816)
positive regulation of DNA-binding transcription factor activity (GO:0051091)
regulation of apoptotic process (GO:0042981)
negative regulation of apoptotic process (GO:0043066)
positive regulation of NF-kappaB transcription factor activity (GO:0051092)
regulation of I-kappaB kinase/NF-kappaB signaling (GO:0043122)
negative regulation of programmed cell death (GO:0043069)
regulation of angiogenesis (GO:0045765)
phosphorylation (GO:0016310)
positive regulation of nucleic acid-templated transcription (GO:1903508)
protein phosphorylation (GO:0006468)
cellular response to oxygen-containing compound (GO:1901701)
regulation of intracellular signal transduction (GO:1902531)
positive regulation of transcription by RNA polymerase II (GO:0045944)
positive regulation of transcription, DNA-templated (GO:0045893)
GO term
300 500 700 900
Combined score
Gene_number
20 30 40 50
log10_Pvalue
20 15
GO enrichment of SS DACs
Fc-epsilon receptor signaling pathway (GO:0038095)
Fc receptor signaling pathway (GO:0038093)
positive regulation of blood vessel endothelial cell migration (GO:0043536)
regulation of blood vessel endothelial cell migration (GO:0043535)
Fc-gamma receptor signaling pathway (GO:0038094)
Fc receptor mediated stimulatory signaling pathway (GO:0002431)
Fc-gamma receptor signaling pathway involved in phagocytosis (GO:0038096)
cellular response to cytokine stimulus (GO:0071345)
cytokine-mediated signaling pathway (GO:0019221)
cellular response to transforming growth factor beta stimulus (GO:0071560)
positive regulation of nucleic acid-templated transcription (GO:1903508)
positive regulation of intracellular signal transduction (GO:1902533)
peptidyl-serine phosphorylation (GO:0018105)
peptidyl-serine modification (GO:0018209)
regulation of I-kappaB kinase/NF-kappaB signaling (GO:0043122)
regulation of apoptotic process (GO:0042981)
Ras protein signal transduction (GO:0007265)
negative regulation of apoptotic process (GO:0043066)
positive regulation of I-kappaB kinase/NF-kappaB signaling (GO:0043123)
antigen receptor-mediated signaling pathway (GO:0050851)
positive regulation of transcription, DNA-templated (GO:0045893)
regulation of angiogenesis (GO:0045765)
positive regulation of protein phosphorylation (GO:0001934)
positive regulation of transcription by RNA polymerase II (GO:0045944)
negative regulation of programmed cell death (GO:0043069)
GO term
200 400 600 800
Combined score
log10_Pvalue
22.5 20.0 17.5 15.0 12.5
Gene_number
20 30 40 50 60 70
GO enrichment of STLMS DACs
pre-replicative complex assembly involved in nuclear cell cycle DNA replication (GO:0006267)
nuclear DNA replication (GO:0033260)
double-strand break repair via break-induced replication (GO:0000727)
DNA replication initiation (GO:0006270)
pre-replicative complex assembly (GO:0036388)
positive regulation of mitochondrial outer membrane permeabilization involved in apoptotic signaling pathway (GO:1901030)
positive regulation of apoptotic signaling pathway (GO:2001235)
DNA replication (GO:0006260)
cell cycle G1/S phase transition (GO:0044843)
G1/S transition of mitotic cell cycle (GO:0000082)
negative regulation of apoptotic process (GO:0043066)
Fc-epsilon receptor signaling pathway (GO:0038095)
Fc receptor signaling pathway (GO:0038093)
DNA-dependent DNA replication (GO:0006261)
peptidyl-serine phosphorylation (GO:0018105)
positive regulation of nucleic acid-templated transcription (GO:1903508)
peptidyl-serine modification (GO:0018209)
cytokine-mediated signaling pathway (GO:0019221)
regulation of apoptotic process (GO:0042981)
negative regulation of programmed cell death (GO:0043069)
inflammatory response (GO:0006954)
positive regulation of intracellular signal transduction (GO:1902533)
cellular response to cytokine stimulus (GO:0071345)
positive regulation of transcription by RNA polymerase II (GO:0045944)
positive regulation of transcription, DNA-templated (GO:0045893)
GO term
0 10000 20000
Combined score
log10_Pvalue
14 12 10 8
Gene_numbe
10 20 30
GO enrichment of ULMS DACs
pre-replicative complex assembly involved in nuclear cell cycle DNA replication (GO:0006267)
nuclear DNA replication (GO:0033260)
double-strand break repair via break-induced replication (GO:0000727)
DNA replication initiation (GO:0006270)
positive regulation of mitochondrial outer membrane permeabilization involved in apoptotic signaling pathway (GO:1901030)
cell cycle G1/S phase transition (GO:0044843)
pre-replicative complex assembly (GO:0036388)
G1/S transition of mitotic cell cycle (GO:0000082)
DNA replication (GO:0006260)
Fc-gamma receptor signaling pathway involved in phagocytosis (GO:0038096)
Fc-gamma receptor signaling pathway (GO:0038094)
Fc receptor mediated stimulatory signaling pathway (GO:0002431)
Ras protein signal transduction (GO:0007265)
positive regulation of cellular metabolic process (GO:0031325)
small GTPase mediated signal transduction (GO:0007264)
DNA-dependent DNA replication (GO:0006261)
cellular response to cytokine stimulus (GO:0071345)
negative regulation of apoptotic process (GO:0043066)
regulation of apoptotic process (GO:0042981)
cytokine-mediated signaling pathway (GO:0019221)
positive regulation of apoptotic process (GO:0043065)
apoptotic process (GO:0006915)
positive regulation of programmed cell death (GO:0043068)
negative regulation of programmed cell death (GO:0043069)
positive regulation of intracellular signal transduction (GO:1902533)
GO term
0 5000 10000 15000 20000
Combined score
log10_Pvalue
14 12 10 8
Gene_number
10 20 30
GO enrichment of UPS DACs
pre-replicative complex assembly involved in nuclear cell cycle DNA replication (GO:0006267)
extrinsic apoptotic signaling pathway in absence of ligand (GO:0097192)
DNA replication initiation (GO:0006270)
positive regulation of mitochondrial outer membrane permeabilization involved in apoptotic signaling pathway (GO:1901030)
Fc-gamma receptor signaling pathway involved in phagocytosis (GO:0038096)
Fc-gamma receptor signaling pathway (GO:0038094)
Fc receptor mediated stimulatory signaling pathway (GO:0002431)
vascular endothelial growth factor receptor signaling pathway (GO:0048010)
extrinsic apoptotic signaling pathway (GO:0097191)
cellular response to cytokine stimulus (GO:0071345)
regulation of apoptotic process (GO:0042981)
cellular response to transforming growth factor beta stimulus (GO:0071560)
transforming growth factor beta receptor signaling pathway (GO:0007179)
cytokine-mediated signaling pathway (GO:0019221)
negative regulation of apoptotic process (GO:0043066)
apoptotic process (GO:0006915)
positive regulation of cysteine-type endopeptidase activity involved in apoptotic process (GO:0043280)
positive regulation of intracellular signal transduction (GO:1902533)
response to cytokine (GO:0034097)
negative regulation of programmed cell death (GO:0043069)
peptidyl-serine phosphorylation (GO:0018105)
positive regulation of apoptotic process (GO:0043065)
positive regulation of programmed cell death (GO:0043068)
transmembrane receptor protein tyrosine kinase signaling pathway (GO:0007169)
regulation of I-kappaB kinase/NF-kappaB signaling (GO:0043122)
GO term
0 5000 10000 15000
Combined score
log10_Pvalue
25 20 15
Gene_number
20 40 60
