# Supplementary figures and images for: Functional Profiling of Soft Tissue Sarcoma Using Mechanistic Models
Source: Int J Mol Sci. 2023 Sep 29;24(19):14732. doi: 10.3390/ijms241914732 (PMC10572617; doi:10.3390/ijms241914732)

(a) tSNE plot of mesenchymal expression data

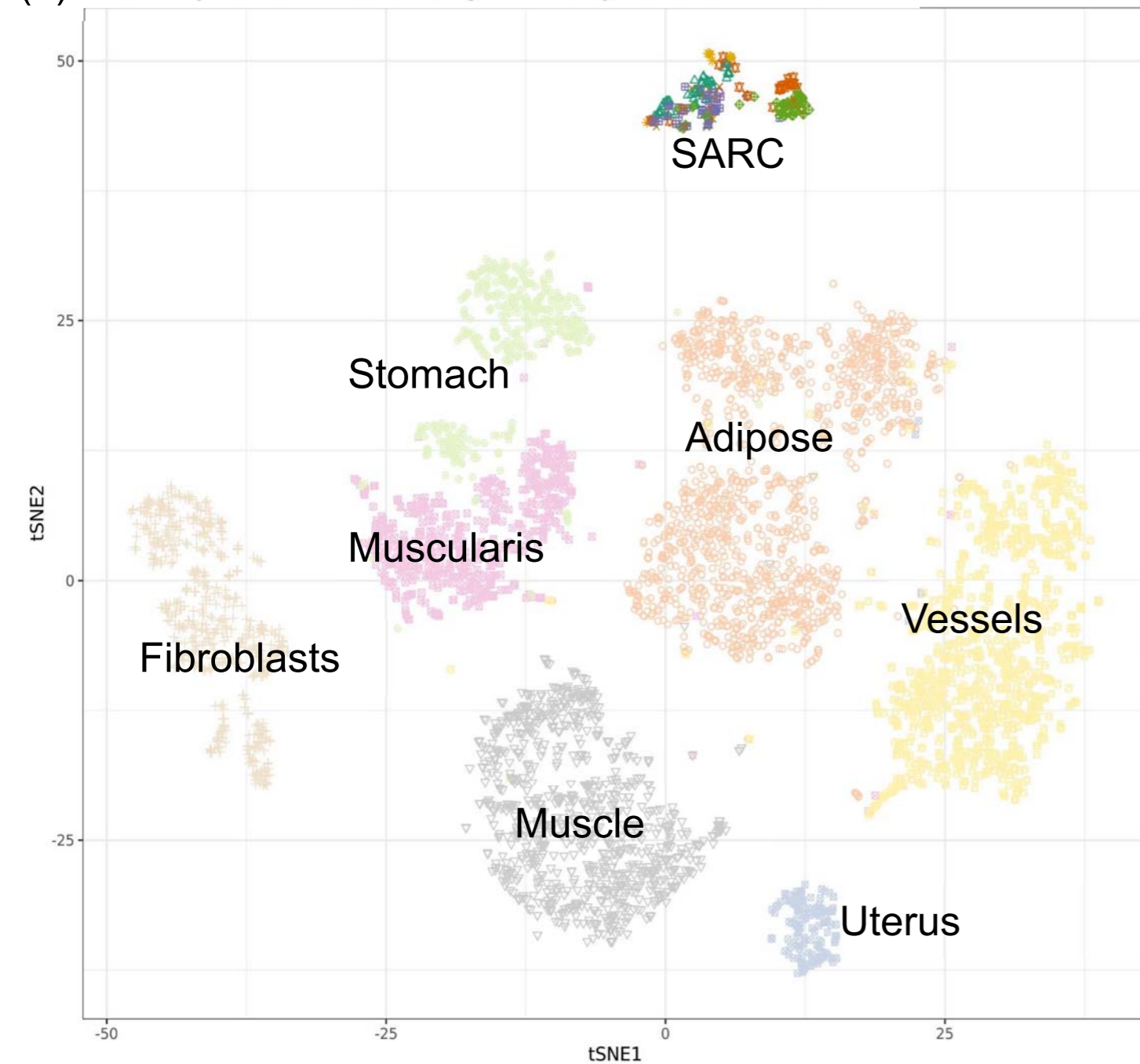

(b) tSNE plot of TCGA sarcoma expression values

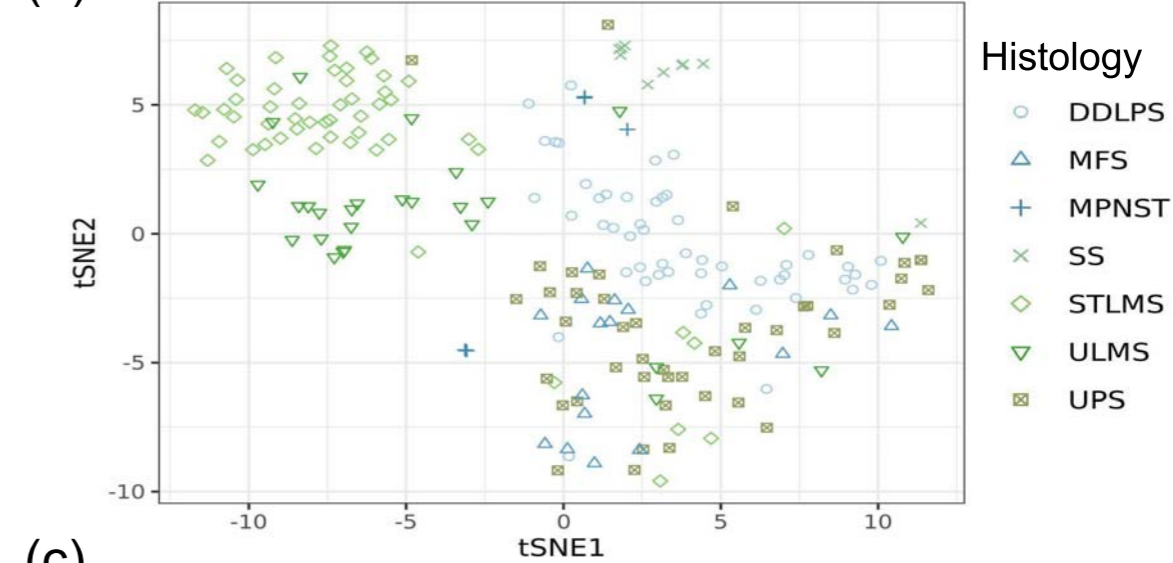

(c)

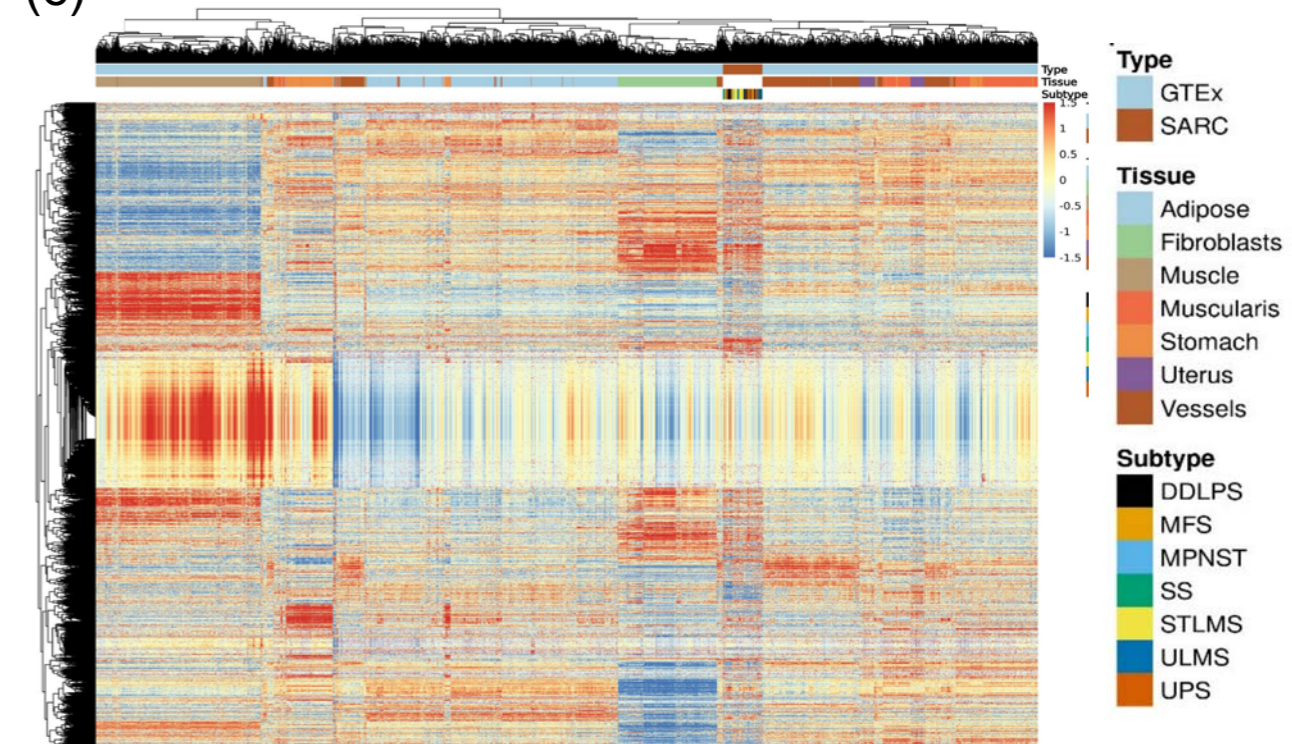

Supplement: Supplementary file 1 [file ijms-24-14732-s001.zip › figure S1.pdf]

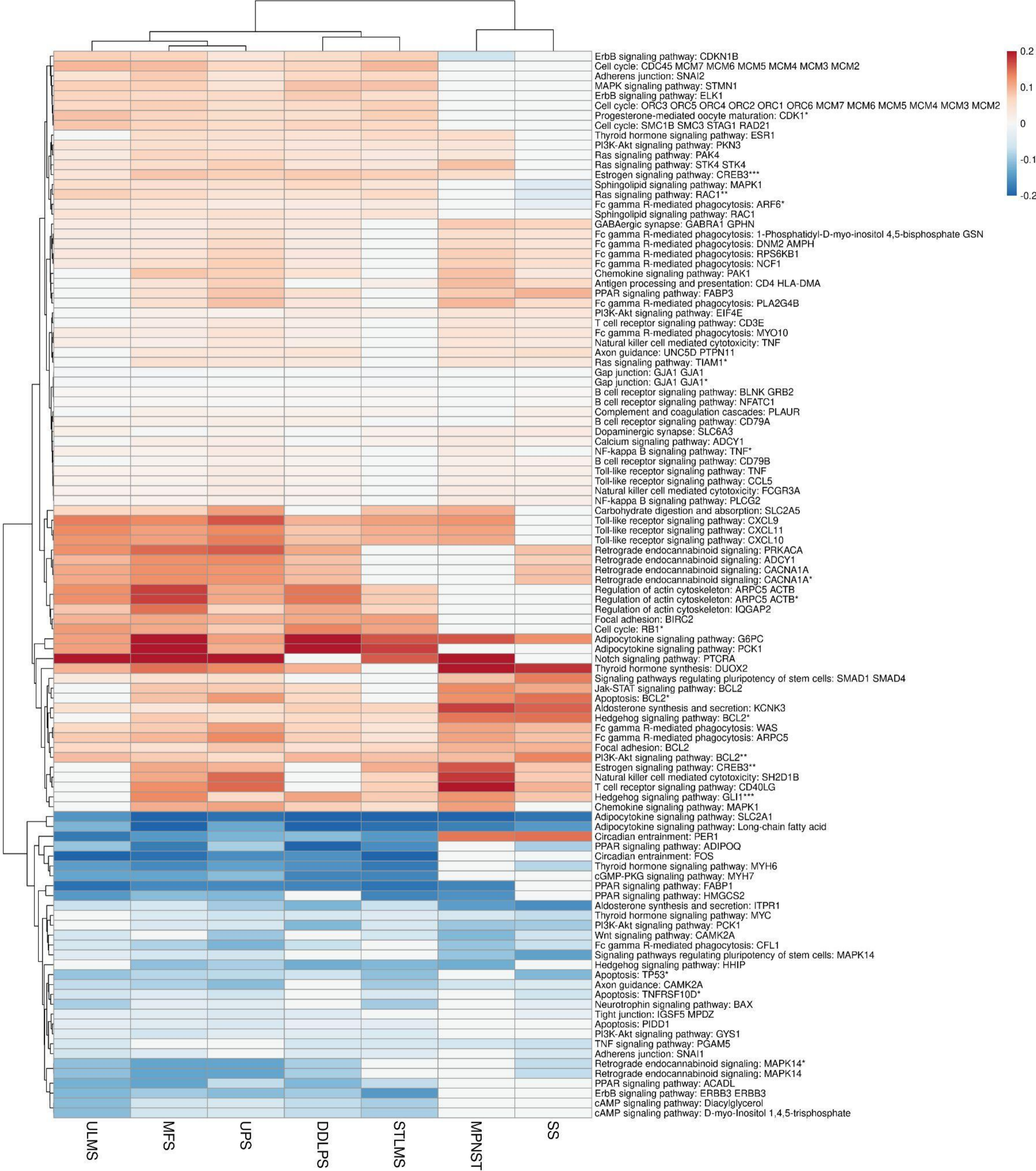

Supplement: Supplementary file 1 [file ijms-24-14732-s001.zip › figure S4.pdf]
